# Fermentation of Soy Milk via *Lactobacillus plantarum* Improves Dysregulated Lipid Metabolism in Rats on a High Cholesterol Diet

**DOI:** 10.1371/journal.pone.0088231

**Published:** 2014-02-10

**Authors:** Yunhye Kim, Sun Yoon, Sun Bok Lee, Hye Won Han, Hayoun Oh, Wu Joo Lee, Seung-Min Lee

**Affiliations:** Department of Food and Nutrition, College of Human Ecology, Yonsei University, Seoul, South Korea; Clermont Université, France

## Abstract

We aimed to investigate whether *in vitro* fermentation of soy with *L. plantarum* could promote its beneficial effects on lipids at the molecular and physiological levels. Rats were fed an AIN76A diet containing 50% sucrose (w/w) (CTRL), a modified AIN76A diet supplemented with 1% (w/w) cholesterol (CHOL), or a CHOL diet where 20% casein was replaced with soy milk (SOY) or fermented soy milk (FSOY). Dietary isoflavone profiles, serum lipids, hepatic and fecal cholesterol, and tissue gene expression were examined. The FSOY diet had more aglycones than did the SOY diet. Both the SOY and FSOY groups had lower hepatic cholesterol and serum triglyceride (TG) than did the CHOL group. Only FSOY reduced hepatic TG and serum free fatty acids and increased serum HDL-CHOL and fecal cholesterol. Compared to CHOL, FSOY lowered levels of the nuclear forms of SREBP-1c and SREBP-2 and expression of their target genes, including FAS, SCD1, LDLR, and HMGCR. On the other hand, FSOY elevated adipose expression levels of genes involved in TG-rich lipoprotein uptake (ApoE, VLDLR, and Lrp1), fatty acid oxidation (PPARα, CPT1α, LCAD, CYP4A1, UCP2, and UCP3), HDL-biogenesis (ABCA1, ApoA1, and LXRα), and adiponectin signaling (AdipoQ, AdipoR1, and AdipoR2), as well as levels of phosphorylated AMPK and ACC. SOY conferred a similar expression profile in both liver and adipose tissues but failed to reach statistical significance in many of the genes tested, unlike FSOY. Our data indicate that fermentation may be a way to enhance the beneficial effects of soy on lipid metabolism, in part via promoting a reduction of SREBP-dependent cholesterol and TG synthesis in the liver, and enhancing adiponectin signaling and PPARα-induced expression of genes involved in TG-rich lipoprotein clearance, fatty acid oxidation, and reverse cholesterol transport in adipose tissues.

## Introduction

Moderate intake of dietary cholesterol has been recommended to avoid undesirable health conditions. High serum cholesterol and/or triglyceride (TG) levels are well-known risk factors for the development of disorders of lipid regulation, including cardiovascular disease [1]. For instance, people with atherosclerosis, a hallmark of cardiovascular disorders, often exhibit hypercholesterolemia, hypertriacylglyceridemia, and/or low serum HDL-CHOL [2]. Dietary cholesterol, along with fatty acids, has been shown to be a significant contributor to blood cholesterol levels [3]. The negative impact of high dietary cholesterol on health appears to be lessened by other food components such as isoflavones or polyphenols [4]. In contrast, a cholesterol-rich diet is more harmful when the diet contains high saturated fatty acids and high sucrose [5], a common feature of diets of Western society.

Previous meta-analyses and animal studies have suggested that soy is a dietary component that can reduce risk factors for diseases associated with dysregulated cholesterol metabolism [4], [6], [7]. Soy proteins rich in isoflavones have been suggested to have protective effects against disturbed serum lipid profiles in animals [6], [8]. Despite considerable data, there have also been conflicting reports, either demonstrating that soy protein isolates lacking isoflavones have a significant effect on blood lipids [9] or alternately demonstrating the necessity of soy isoflavones for beneficial effects [4], [8]. It is possible that the effects of separate components of soy may be synergistic, producing greater effects in concert than they do as isolated components [7]. Additionally, differences in the bioavailability of soy constituents could influence the efficacy of soy products. The majority of naturally occurring soy isoflavones exists in the form of conjugated glucosides that have undergone modifications such as malonylation and acetylation. *In vitro* fermentation by probiotics has been shown to increase the bioavailability of isoflavones by removing glycosyl moieties and transforming them into aglycone structures [10]. Soy isoflavone aglycones are absorbed more easily by the intestine [11]. Therefore, fermentation may increase the efficacy of soy products by enhancing the intestinal bioavailability of soy constituents.

Serum lipid metabolism is tightly regulated, in part through modulation of hepatic gene expression [12]. At the molecular level, the sterol regulatory element binding protein (SREBP)-1 transcribes genes required for fatty acid synthesis, thus increasing TG, while SREBP-2 governs cholesterol metabolism by regulating the expression of its target genes, including low density lipoprotein receptor (LDLR) and 3-hydroxy-3-methylglutaryl-CoA reductase (HMGCR) [12]. LDLR is a receptor for LDL-CHOL, and HMGCR is a rate-limiting enzyme in cholesterol biosynthesis; their expression ultimately increases intracellular cholesterol levels [12]. In addition to SREBP-mediated transcriptional regulation, other transcription factors, such as liver X receptor alpha (LXRα) [13], and/or various regulatory steps at the post-transcriptional and/or post-translational level are also important for regulation of lipid metabolism [14], [15].

Although there have been numerous studies on soy protein or soy constituents, the effects of fermented whole soy products on the dysregulation of lipid metabolism and gene expression resulting from a high cholesterol diet have not been studied at the molecular level [16], [17]. The objectives of the current study were to examine the efficacy of soy milk fermentation in facilitating its beneficial effects on serum lipids, and to investigate novel molecular mechanisms of whole soy milk as a serum lipid-based health-improving food source. To achieve this, we first investigated soy isoflavone profiles in both SOY and FSOY and then evaluated the effects of SOY and FSOY on serum lipids and hepatic and fecal lipid content in rats fed a high cholesterol diet. Subsequent evaluation of their effects on gene expression in liver and adipose tissues was also performed. Molecular analyses of the effects of SOY and FSOY may allow us to further define the impact of fermentation on lipid metabolism, thereby providing an opportunity to explore the use of fermentation by lactic acid bacteria to maximize the beneficial effects of soy products.

## Materials and Methods

### Animals

Thirty-two five-week-old male Sprague Dawley rats (Koatech, Pyungtek, Korea) were divided into four experimental groups (CTRL, CHOL, SOY, and FSOY) of eight animals each. Each group was fed an experimental diet *ad libitum* for six weeks (CTRL: AIN76A diet; CHOL: 1% high cholesterol-containing AIN76A diet; SOY: modified AIN76A diet in which 20% casein and 40% corn oil were replaced with freeze-dried SOY; FSOY: modified AIN76A diet in which 20% casein and 40% corn oil were replaced with freeze-dried FSOY) (Table 1). All animals were fed experimental diets daily and housed in a pathogen-free environment with controlled temperature and humidity (18–24°C, 50–60%, respectively). Rats were fasted for 12 h prior to sacrifice and were euthanized with diethyl ether. Tissues were dissected, snap-frozen in liquid nitrogen, and then stored at −80°C until use. For analysis of blood lipids, blood samples were collected through the abdominal inferior vena cava. Serum was obtained by centrifugation at 3,000 rpm for 20 min and stored in a freezer at −80°C until analysis. Feces from each group were collected for three days prior to sacrifice. All experimental procedures were reviewed and approved by the Committee on Animal Experimentation and Ethics of Yonsei University (YLARC Permit #: 2010-0044).

**Table 1:** Diet compositions (g ingredient/kg diet) in the experimental groups.

	Diet Groups			
Ingredients	CTRL^1^	CHOL^2^	SOY^3^	FSOY^4^
Sucrose	499.99	499.99	461.59	461.59
Corn starch	150	150	138.3	138.3
Casein	200	200	160	160
Soy milk/Fermented soy milk	–	–	124.2	124.2
Corn oil	50	50	28.8	28.8
Mineral mix^5^	35	35	35	35
Vitamin mix^6^	10	10	10	10
Cellulose	50	50	50	50
DL-methionine	3	3	3	3
Choline Bitartrate	2	2	2	2
Ethoxyquin	0.01	0.01	0.01	0.01
Cholesterol	–	10	10	10
Cholic acid	–	5	5	5
Total	1000	1015	1027.9	1027.9

1 Control AIN76A diet,

2 High cholesterol diet,

3 Soy milk diet,

4 Fermented soy milk diet,

5 AIN-93 mineral mix, and

6 AIN-93 vitamin mix.

### Preparation of Diets Containing Freeze-dried SOY and FSOY

SOY was produced by Yonsei Milk (Asan, Korea). Soy milk, derived from whole soy, contained 5 g of carbohydrate, 7 g of protein, 3.5 g of lipids, 2 g of dietary fiber, and 150 mg of sodium per kg. To produce FSOY, 0.1% (w/v) *Lactobacillus plantarum* KCTC10782BP (Cell Biotech International Korea, Seoul, Korea) was used. After the addition of 20 g of Galacto-oligosaccharide (Samyang, Seoul, Korea), fermentation proceeded at 25°C until the acidity reached pH 5.1. After being filtered, SOY and FSOY were freeze-dried (ILShinBioBase, Dongduchen, Korea).

### Determination of Isoflavone Content

In brief, 1 g of freeze-dried SOY and FSOY diets were mixed with 25% methanol (1∶4, v/v) containing 400 µg of caffeine as an internal standard at 25°C for 1 h with sonication and then centrifuged to remove the insoluble fraction. Each supernatant was filtered through a 0.45 µm GHP column (PALL Life Sciences, Port Washington, NY, USA). The filtrate was analyzed using ultraperformance liquid chromatography (UPLC) (Waters, Palo Alto, CA, USA) with a cyano column (2.1 mm × 50 mm, 1.8 µm, HSS Cyano; Waters). The mobile phases were water containing 0.1% formic acid and acetonitrile containing 0.1% formic acid. The temperature of the column was 30°C and chromatograms were recorded at 260 nm. Daidzein, daidzin, genistein, and genistin were identified based on their retention times after comparison with corresponding standards purchased from Sigma Aldrich (St. Louis, MO, USA).

### Determination of Isoflavone Derivatives by Mass Spectrometry

LC-MS identification was performed using UPLC (Acquity UPLC™; Waters, Milford, MA, USA) and LTQ Orbitrap (Thermo Fisher Scientific, Waltham, MA, USA), equipped with ESI. Data analysis was performed with MassLynx™ 4.0 data system software (Waters).

### Determination of Hepatic and Fecal Lipids, Blood Lipid Profiles, and Fecal Bile Acid

Lipids from liver and feces were extracted using a modified Folch method [18]. The isolated lipid fraction was used to measure TOTAL-CHOL and TG concentrations using commercially available kits (Asan Pharm, Seoul, Korea). Serum TOTAL-CHOL, HDL-CHOL, and TG levels were also measured with commercially available kits (Asan Pharm, Seoul, Korea). All determinants were obtained by absorbance at 550 nm using a microplate reader (Molecular Device, Sunnyvale, CA, USA). LDL-CHOL was calculated using the Friedewald formula [19] (LDL-CHOL (mg/dl) = TOTAL-CHOL *–* HDL-CHOL *–* TG/5). Atherogenic indices were obtained by the Haglund method (Atherogenic index = (TOTAL-CHOL *–* HDL-CHOL)/HDL-CHOL) [20]. Bile acid was extracted using the DeWael method [21]. Extracted fecal bile acid was measured using a kit from Bioquant (Nashville, TN, USA). Serum glucose levels were measured using the Fuji DRI-CHEM 4000i analyzer (Fuji Film Co., Tokyo, Japan).

### RNA Extraction and Quantitative RT-PCR

Total RNA was extracted from tissues using Trizol according to the manufacturer’s protocol (Invitrogen, Carlsbad, CA, USA). Total RNA (1 µg) was used to synthesize cDNA with ImProm-II reverse transcriptase (Promega, Madison, WI, USA). Real-time PCR was performed on a CFX96 sequence detection system (Bio-Rad, Hercules, CA, USA) using EvaGreen qPCR Mix Plus (Solis BioDyne, Estonia). Expression levels were normalized to the amount of 18S rRNA. Relative mRNA levels were then calculated by the difference in *C_t_* values among animal groups, expressed as the fold change.

### Western Blot Analysis

Tissues were homogenized in RIPA buffer and then centrifuged at 11,000 × *g* for 10 min at 4°C. Equal amounts of protein lysates were loaded on an SDS-polyacrylamide gel. Anti-SREBP-2, anti-SREBP-1c, and anti-adiponectin antibodies from Santa Cruz Biotechnology (Santa Cruz, CA, USA), anti-p-AMP-activated protein kinase (AMPK) and anti-p-Acetyl CoA carboxylase (ACC) antibodies from Cell Signaling Technology (Beverly, MA, USA), anti-LDLR antibody from BioVision (Milpitas, CA, USA), and anti-GAPDH antibody from Signalway Antibody (College Park, MD, USA) were used to detect the respective proteins.

### Statistical Analyses

SPSS was used to perform statistical analyses (Statistical Package for the Social Sciences; SPSS Inc., Chicago, IL, USA). Student’s *t*-test and one-way analysis of variance (ANOVA) followed by Duncan’s multiple comparisons test were used to determine statistically significant differences among the experimental groups. A *P* value <0.05 was the criterion for statistical significance.

## Results

### FSOY had a Higher Proportion of unmodified Soy Isoflavones and their Aglycones than SOY

Soy isoflavone composition was compared between SOY and FSOY in terms of percentages (Table 2). Fermentation lowered the amounts of both malonyl and acetyl groups in isoflavones. Interestingly, equol was below the detection limit in SOY but represented 0.03% of the total isoflavones in FSOY. In addition, soy isoflavone aglycones, including genistein, daidzein, and glycitein, were increased in FSOY compared to levels in SOY. These results indicate that fermentation increased the proportion of soy isoflavone aglycones as well as equol in FSOY and diminished soy isoflavone glycones conjugated with malonyl and acetyl residues relative to those in SOY.

**Table 2:** Percentage of soy isoflavones in SOY and FSOY^1^.

	SOY	FSOY	*P* 2
Malonyl daidzin	9.27±0.06	0.29±0.01	<0.05
Acetyl daidzin	1.07±0.02	0.43±0.00	<0.05
Daidzin	22.42±0.07	33.14±0.26	<0.05
Daidzein	0.10±0.01	0.72±0.02	<0.05
Equol	0.00±0.00	0.03±0.00	<0.05
Malonyl glycitin	0.8±0.01	0.00±0.00	<0.05
Acetyl glycitin	0.77±0.00	0.00±0.00	<0.05
Glycitin	2.93±0.01	3.73±0.04	<0.05
Glycitein	0.02±0.00	0.09±0.00	<0.05
Malonyl genistin	19.20±0.12	0.33±0.01	<0.05
Acetyl genistin	2.49±0.01	0.89±0.01	<0.05
Genistin	39.85±0.13	58.72±0.30	<0.05
Genistein	1.08±0.01	1.64±0.01	<0.05
Total	100±0.00	100±0.00	

1 Values are presented as mean ± SD obtained from triplicate sample analysis,

2 *P*-value refers to the Student’s *t-*test.

### FSOY Lowered Hepatic Lipids and Serum TG and FFAs, and Elevated HDL-CHOL

**Figure 1:**
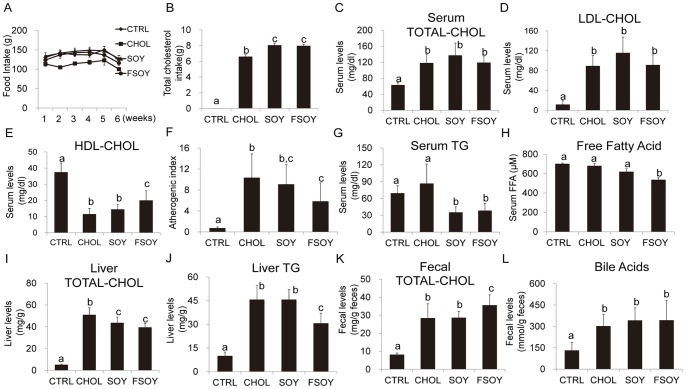
Food intake and lipid profiles of rats fed SOY or FSOY as opposed to CHOL. Food intake (A), total cholesterol intake (B), serum levels of TOTAL-CHOL (C), LDL-CHOL (D), and HDL-CHOL (E), atherogenic index (F), serum levels of TG (G) and free fatty acids (H), hepatic TOTAL-CHOL (I), hepatic TG (J), fecal TOTAL-CHOL (K), and fecal bile acid (L) in each animal group. The results are expressed as means ± SD of eight animal tissues in each group. Values not sharing the same letter were significantly different (*P*<0.05) between all groups by ANOVA.

When fed *ad libitum*, the CHOL group showed a modest decrease in food intake compared to the other groups (Fig. 1A and B). The CHOL, SOY, and FSOY groups demonstrated similar levels of serum TOTAL-CHOL and LDL-CHOL as the CTRL group (Fig. 1C and D), suggesting that SOY and FSOY failed to lower serum TOTAL-CHOL or LDL-CHOL. FSOY significantly restored the CHOL-driven reduction of serum HDL-CHOL, which was not observed with SOY (Fig. 1E). This led to a noticeable reduction of atherogenic index values in the FSOY group compared to the CHOL group (Fig. 1F). Compared to the CHOL diet, both SOY and FSOY lowered hepatic TOTAL-CHOL, although only FSOY increased fecal TOTAL-CHOL, which was not accompanied by a change in fecal bile acid content (Fig. 1I, K, and L). On the other hand, only FSOY significantly lowered CHOL-elevated hepatic TG levels and serum FFAs (Fig. 1J and H). CHOL did not affect serum TG levels, but SOY and FSOY greatly decreased serum TG, to below that of the CTRL group (Fig. 1G). Overall, our data suggest that, despite the higher dietary cholesterol intake due to *ad libitum* feeding, SOY and FSOY did not worsen serum or liver lipid profiles relative to CHOL. On the contrary, SOY and FSOY decreased serum TG and CHOL-elevated hepatic TOTAL-CHOL. Moreover FSOY reduced hepatic TG and serum FFAs, and increased the levels of serum HDL-CHOL.

### FSOY Downregulated Expression of Genes Involved in Lipid Metabolism in the Liver

**Figure 2:**
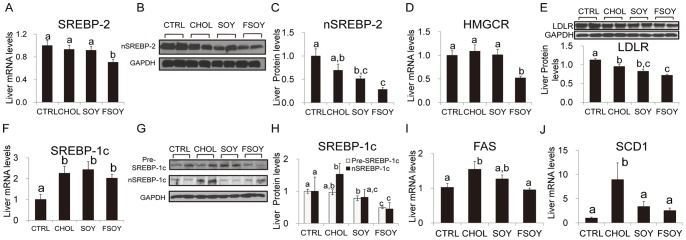
Hepatic expression levels of genes involved in cholesterol and TG metabolism in rats on SOY or FSOY diet compared with CHOL diet. Expression levels of SREBP-2 mRNA (A), nuclear form of SREBP-2 protein (B and C), HMGCR mRNA (D), LDLR protein (E), SREBP-1c mRNA (F), premature and nuclear forms of SREBP proteins (pre-SREBP and nSREBP)(G and H), FAS mRNA (I), and SCD1 mRNA (J). Representative band images indicating protein levels for SREBP-2 (B), SREBP-1c (G), and LDLR (E) are presented. Bands were quantified, normalized, and presented as a graph (C, D, and F). The results are expressed as means ± SE of eight animal tissue samples. Values not sharing the same letter were significantly different (*P*<0.05) between all groups by ANOVA.

Next, we analyzed whether a significant reduction of hepatic cholesterol and TG by FSOY is associated with downregulation of genes involved in lipid metabolism. As for cholesterol metabolism, only FSOY showed a significant reduction in SREBP-2 mRNA and protein compared to levels in the CHOL group (Fig. 2A–C), which was accompanied by a reduction in HMGCR mRNA and LDLR protein (Fig. 2D and E). However, the reduction of LDLR mRNA in the FSOY group was not great enough to reach statistical difference between FSOY and CHOL (data not shown). On the other hand, CHOL increased hepatic transcripts of SREBP-1c, fatty acid synthase (FAS), and stearoyl CoA desaturase (SCD1) relative to CTRL (Fig. 2F, I, and J). FSOY downregulated the mature and nuclear forms of SREBP-1c protein, and its target genes, including FAS and SCD-1, compared to levels in the CHOL group (Fig. 2G–J). SOY and FSOY did not lower the CHOL-elevated SREBP-1c mRNA (Fig. 2F), These data suggest that FSOY might lower hepatic cholesterol and TG in the liver in part through downregulating SREBP-dependent gene expression.

### FSOY Increased Expression of Genes Involved in TG-rich Lipoprotein Clearance and Fatty Acid Oxidation

**Figure 3:**
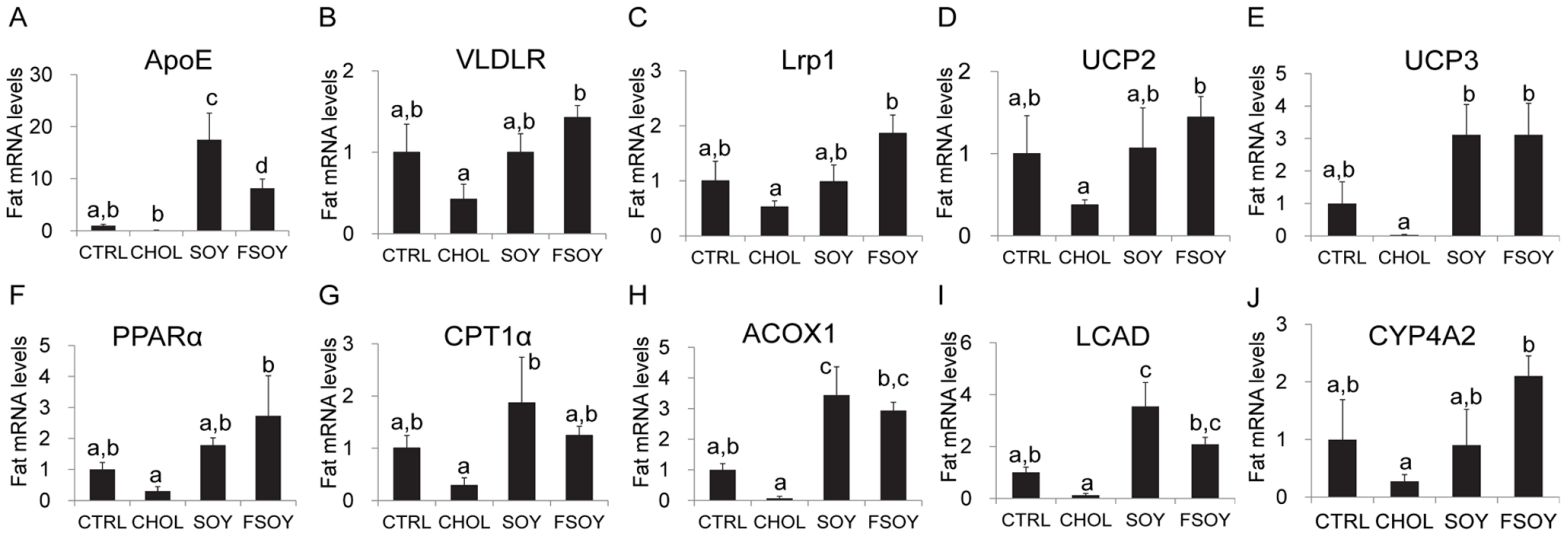
Adipose expression levels of genes related to lipoprotein uptake and fatty acid oxidation in SOY and FSOY groups compared to CTRL and CHOL groups. mRNA levels of ApoE (A), VLDLR (B), Lrp1 (C), UCP2 (D), UCP3 (E), PPARα (F), CPT1α (G) ACOX1 (H), LCAD (I) and CYP4A1 (J) in rats fed experimental diets. The results are expressed as means ± SE of eight animal tissues. Values not sharing the same letter were significantly different (*P*<0.05) between all groups by ANOVA.

To gain insight into the molecular mechanisms of FSOY-lowered serum TG levels, adipose gene expression levels were compared among the groups. ApoE mRNA was significantly increased by both SOY and FSOY (Fig. 3A). However, only FSOY demonstrated a significant increase in mRNA levels of very low density lipoprotein receptor (VLDLR) and low density lipoprotein receptor-related protein 1 (Lrp1) relative to levels in CHOL (Fig. 3B and C). Lipoprotein lipase (LPL) mRNA levels were not affected by any diet (data not shown). On the other hand PPARα, a master transcription factor for fatty acid oxidation, and its target genes, including mitochondrial uncoupling protein 2 (UCP2), UCP3, acyl-CoA oxidase 1, palmitoyl (ACOX1), long-chain specific acyl-CoA dehydrogenase (LCAD), and cytochrome P450 family 4 subfamily a polypeptide 2 (CYP4A2) were significantly upregulated by FSOY, relative to CHOL (Fig. 3 F, H–J). SOY also upregulated some PPAR target genes, such as UCP3, ACOX1, and LCAD (Fig. 3 E, H, and I). Only SOY significantly statistically increased transcript levels of carnitine palmitoyltransferase 1 alpha (CPT1α) compared to levels in the CHOL group (Fig. 3G). These results indicate that CHOL-suppressed fatty acid oxidation genes were partly upregulated by SOY, but that FSOY had a greater effect, on more genes. Only FSOY significantly upregulated the ApoE, VLDLR, and Lrp1 genes, which are involved in TG-rich lipoprotein uptake.

### FSOY upregulated Genes Involved in Reverse Cholesterol Transport

**Figure 4:**
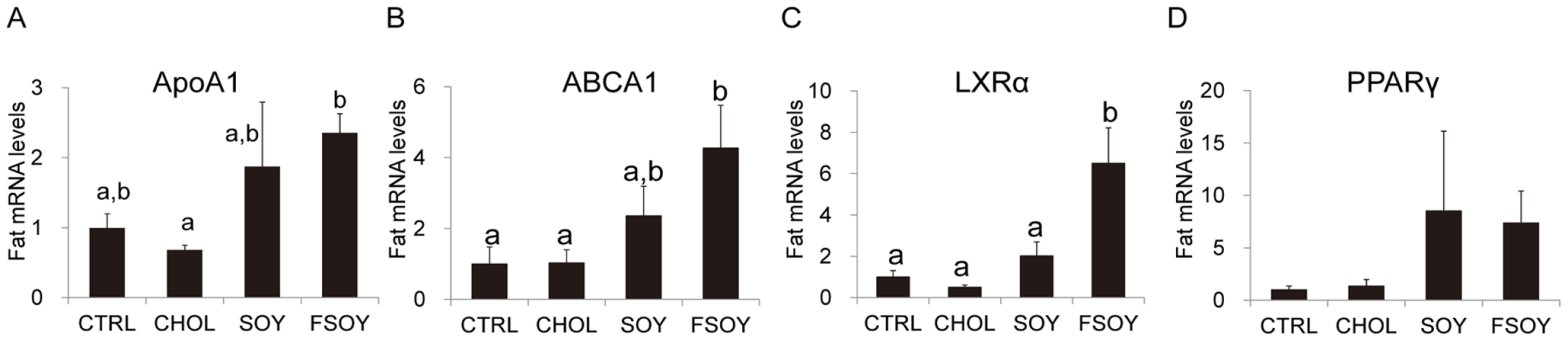
The effects of SOY or FSOY on the mRNA levels of genes involved in reverse cholesterol transport in rats on a high cholesterol diet. Adipose mRNAs of ApoA1 (A), ABCA1 (B), LXRα (C), and PPARγ (D) in rats fed experimental diets. The results are expressed as means ± SE of eight animal tissues. Values not sharing the same letter were significantly different (*P*<0.05) between all groups by ANOVA.

ApoA1 and ATP-binding cassette, sub-family A, member 1 (ABCA1) genes play crucial roles in the formation of HDL-CHOL. FSOY significantly increased transcript levels of ApoA1 and ABCA1 relative to levels in the CHOL group (Fig. 4A and B). LXRα, a transcription factor for ABCA1, was also significantly elevated only by FSOY (Fig. 4C). mRNA levels of peroxisome proliferator activated receptor gamma (PPARγ), a transcription factor for LXRα, did not show any statistical difference among the groups (Fig. 4D). These results indicate that FSOY increased adipose expression levels of genes for reverse cholesterol transport, probably leading to FSOY elevation of serum HDL-CHOL.

### FSOY Increased Expression of Adiponectin Gene and AMPK Signals

**Figure 5:**
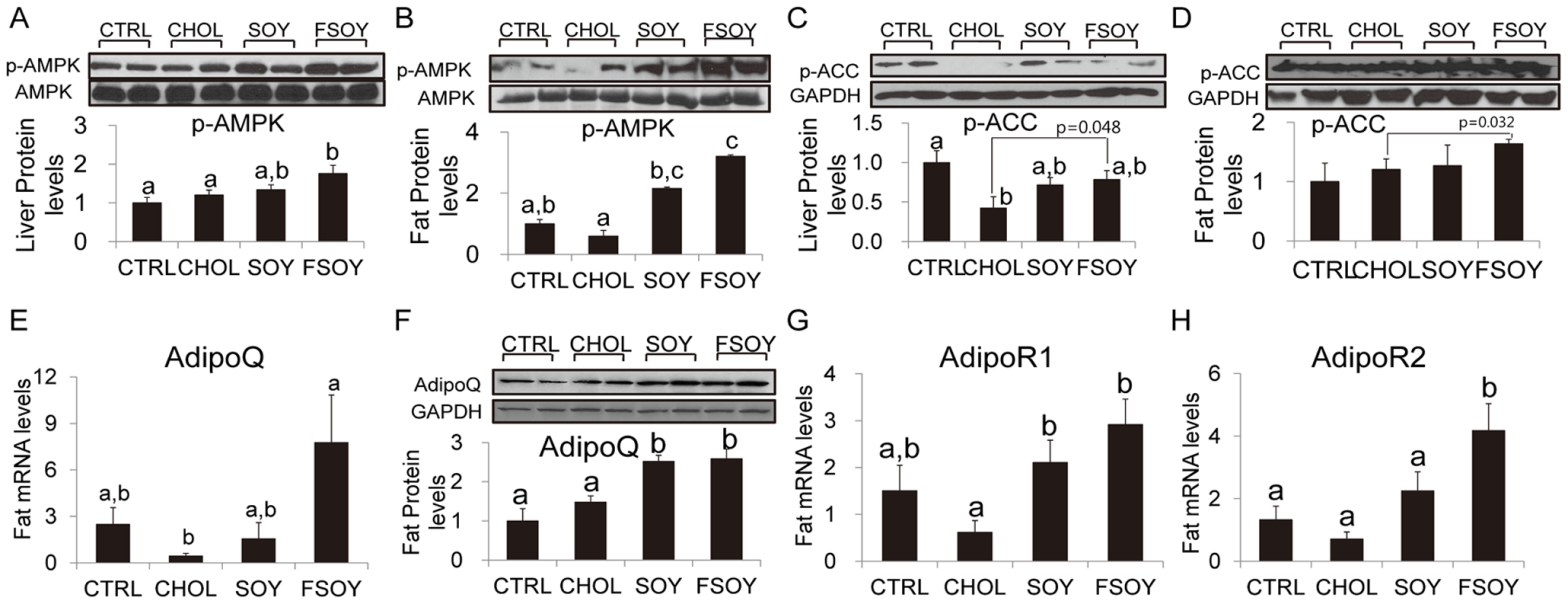
The effects of SOY or FSOY on mRNA levels of adiponectin and adiponectin receptors, and on AMPK activation. Hepatic and adipose levels of phosphorylated AMPK (A and B) and phosphorylated ACC (C and D), fat adiponectin mRNA (E) and protein (F), and mRNA levels of adiponectin receptor 1 (G) and adiponectin receptor 2 (H). The results are expressed as means ± SE of eight animal tissues. Values not sharing the same letter were significantly different (*P*<0.05) between all groups by ANOVA, and Student’s *t*-test was performed to compare the hepatic and adipose protein levels of p-ACC between the CHOL and FSOY groups.

Compared to CHOL, FSOY increased mRNA and protein levels of AdipoQ and transcript levels of the AdipoR1 and -R2 genes in adipose tissue, whereas SOY resulted in a significant increase only in AdipoQ protein and AdipoR1 mRNA (Fig. 5A–D). Phosphorylation of AMPK, a target of adiponectin signaling, was elevated in the liver and adipose tissues by both SOY and FSOY compared to CHOL, but with greater statistical significance by FSOY than by SOY (Fig. 5E and F). ACC, an enzyme involved in fatty acid synthesis, is inactivated by AMPK-mediated phosphorylation [22]. FSOY increased the levels of p-ACC in the liver and adipose tissues (Fig. 5C and D). Our data indicate that FSOY is likely to increase adiponectin signals as well as AMPK activation.

## Discussion

In the present study we provided evidence demonstrating that beneficial effects of soy milk on lipid metabolism in rats on a high cholesterol and high sucrose diet were further augmented by fermentation of soy milk. *L. plantarum* fermentation of soy milk resulted in modification of the isoflavones in soy milk, increasing deconjugated and aglycone forms of isoflavones. As expected from a previous report showing that *L. plantarum* has strong β-glucosidase activity [23], FSOY not only had a greater proportion of aglycones, but also glycones without malonyl and acetyl groups than did SOY. These chemical changes in the soy isoflavone components of FSOY are likely to contribute to the specific effects of FSOY by increasing the amount of bioavailable soy isoflavones compared to those found in SOY. In fact, a human study demonstrated that *in vivo* bioavailability of isoflavones is enhanced by fermentation, compared to untreated soy milk [17]. The fermentation-promoted beneficial effects of soy milk on lipid metabolism were demonstrated by FSOY-specific reduction of hepatic TG, serum FFAs, and atherogenic indices, and an FSOY-specific increase in serum HDL-CHOL in addition to a decrease in hepatic cholesterol and serum TG as shown in SOY, compared to CHOL.

In order to understand the molecular mechanisms that mediate these beneficial effects of fermented soy milk, we evaluated the expression levels of hepatic and adipose genes related to lipid metabolism. Not only SREBP-2, a master transcription factor governing increases in cellular cholesterol levels, but also the SREBP-2 target genes, HMGCR and LDLR, were significantly downregulated only by FSOY. FSOY-specific significant downregulation of SREBP-2 and its target genes would contribute to the greater reduction of hepatic cholesterol levels relative to that conferred by SOY. Our data regarding FSOY effects on SREBP-2 expression levels are not consistent with a previous report showing that a soy protein diet increases SREBP-2 expression levels [24]. This discrepancy might have arisen from the fact that we used whole soy milk containing many other soy components, including fatty acids and/or isoflavones. In addition, SREBP-1c, a key transcription factor involved in the synthesis of fatty acids, and its target genes, FAS and SCD1, were significantly downregulated by FSOY. SREBP-1c increases the transcription of FAS and SCD-1, resulting in an increase in the synthesis of TG [12]. CHOL induced SREBP-1c gene expression and processing, resulting in an increase in the nuclear form of SREBP-1c protein, which is consistent with the marked elevation of hepatic TG in CHOL. Thus FSOY-induced downregulation of SREBP-1c may lower CHOL-increased hepatic TG. SOY demonstrated similar effects in its regulation of SREBP-1c, but they were not strong enough to lower hepatic TG relative to that in CHOL with statistical significance. In addition, FSOY increased phosphorylated AMPK levels in the liver. AMPK phosphorylates and inactivates ACC and HMGCR [25], thus the AMPK activation observed in FSOY may further decrease synthesis of fatty acids and cholesterol by inhibiting the catalytic enzyme activities of ACC and HMGCR, respectively. Taken together, the downregulation of expression and processing of SREBP genes and of the expression of their target genes, as well as of AMPK activation, appear to contribute to the reduction of hepatic lipids in the FSOY group.

Hepatic cholesterol levels could also be affected by decreased dietary cholesterol absorption. FSOY increased fecal cholesterol levels without lowering fecal bile acid contents, suggesting that FSOY might confer decreased dietary cholesterol absorption instead of increasing biliary cholesterol excretion. According to our unpublished data, *L. plantarum* bound to cholesterol lowers its solubility in *in vitro* experiments, suggesting that fermented products containing lactic acids could reduce the bioavailability of dietary cholesterol for intestinal absorption by lowering the solubility of cholesterol in the intestine.

Upregulation of ApoA1, ABCA1, and liver X receptor α (LXRα) was detected only in the FSOY group. ApoA1 is an apolipoprotein found in nascent HDL-CHOL, and the ABCA1 transporter incorporates cholesterol and phospholipids into lipid-poor HDL particles [26]. FSOY-specific upregulation of ApoA1 and ABCA1 genes may result in an increase in ApoA1-dependent cholesterol efflux via ABCA1 in adipose tissue in HDL-CHOL biogenesis. A significant elevation of liver X receptor α (LXRα) mRNA found in the FSOY group could mediate ABCA1 upregulation, given that the nuclear receptors LXRα and/or LXRβ, and retinoid X receptor RXR, activate ABCA1 expression [27]. On the other hand, we did not observe significant changes in serum LDL-CHOL even though it has been reported in a meta-analysis that the consumption of soy isoflavones lowers serum LDL-CHOL [4]. It is possible that the replacement of 20% of dietary protein with SOY or FSOY might not have been sufficient to elicit a serum LDL-CHOL-lowering effect.

FSOY-driven serum TG reduction may be related to an increase in the uptake and catabolism of TG-rich lipoproteins in peripheral tissues. The transcript levels of ApoE, an apolipoprotein found in TG-rich lipoproteins, VLDLR, a receptor for apoE-containing VLDL, and Lrp1, an apoE-receptor protein, were increased in the FSOY group. Lack of VLDLR has been demonstrated to elevate TG content in circulating serum [28]. Lrp1 mRNA levels have been shown to be positively associated with levels of serum TG clearance [29]. FSOY may promote the oxidation of fatty acids taken up from TG-rich lipoproteins and remove them by dissipating heat through the respiratory uncoupling process. In our study, FSOY upregulated fatty acid oxidation-related genes, including PPARα and its target genes, such as ACOX1, LCAD, and CYP4A1, which encode products involve in various types of fatty acid oxidation processes such as peroxisomal, mitochondrial, and microsomal oxidation, respectively, as well as UCP2 and UCP3, which dissipate energy as heat [30]. The roles of UCP proteins in the control of serum TG were demonstrated by a marked reduction of blood TG in transgenic mice overexpressing adipose tissue-specific Ucp1 [31]. Thus FSOY-induced increases in fatty acid oxidation and the respiratory uncoupling process in adipose tissue could lead to a significant clearance of serum TG. At the same time the FSOY effects on adipose fatty acid oxidation may also be enhanced by increased activation of the adiponectin signaling pathway. PPARα activity is facilitated by adiponectin signaling [32]. FSOY increased the expression of AdipoQ, AdipoR1, and AdipoR2, which could enhance adiponectin signaling and promote PPARα activity. Furthermore, FSOY-induced phosphorylation of AMPK and ACC may have promoted fatty acid oxidation in the FSOY group. ACC is an enzyme that generates malonyl-CoA, which inhibits fatty acid influx into mitochondria for β-oxidation [22]. Activated AMPK phosphorylates ACC, resulting in a decrease in the production of malonyl-CoA, thereby permitting an influx of fatty acids into mitochondria for β-oxidation [22]. Additionally, FSOY-induced reduction of serum FFAs may lower the production of TG rich-VLDL by the liver, given that an elevation of serum FFA levels increases the influx of FFA into the liver for VLDL production [33]. Overall, FSOY-induced upregulation of genes involved in TG-rich lipoprotein uptake and fatty acid oxidation, as well as AMPK activation, may increase serum TG clearance.

**Figure 6:**
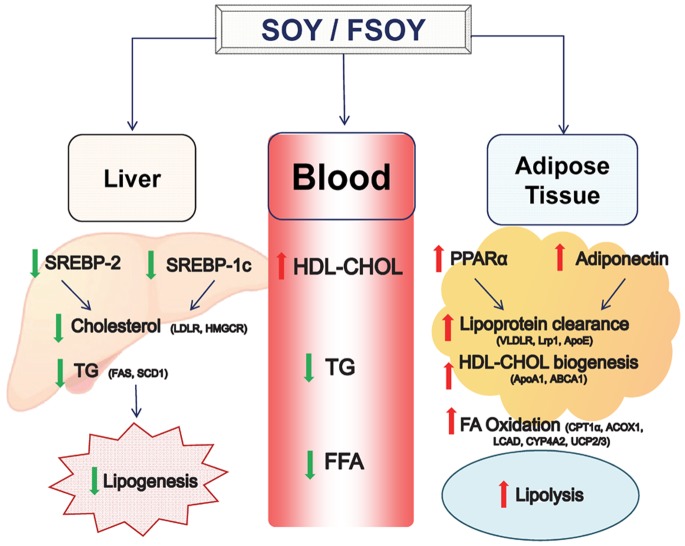
Proposed model of the beneficial effects of fermented soy on lipid profiles and related gene expression in the liver and adipose tissues of rats on a high cholesterol diet.

In the present study we provide mechanistic evidence supporting the beneficial effects of soy milk in terms of regulation of expression of genes in lipid metabolism (Fig. 6). In addition, we suggest that fermentation might be an effective way to increase the beneficial effects of soy on the dysregulation of lipid metabolism induced by a high cholesterol diet.
